# The Impact of Cannabidiol on Human Brain Function: A Systematic Review

**DOI:** 10.3389/fphar.2020.618184

**Published:** 2021-01-21

**Authors:** Albert Batalla, Julian Bos, Amber Postma, Matthijs G. Bossong

**Affiliations:** Department of Psychiatry, UMC Utrecht Brain Center, Utrecht University, Utrecht, Netherlands

**Keywords:** cannabidiol, delta9-tetrahydrocannabinol, *Cannabis* (marijuana), neuroimaging, functional MRI

## Abstract

**Background:** Accumulating evidence suggests that the non-intoxicating cannabinoid compound cannabidiol (CBD) may have antipsychotic and anxiolytic properties, and thus may be a promising new agent in the treatment of psychotic and anxiety disorders. However, the neurobiological substrates underlying the potential therapeutic effects of CBD are still unclear. The aim of this systematic review is to provide a detailed and up-to-date systematic literature overview of neuroimaging studies that investigated the acute impact of CBD on human brain function.

**Methods:** Papers published until May 2020 were included from PubMed following a comprehensive search strategy and pre-determined set of criteria for article selection. We included studies that examined the effects of CBD on brain function of healthy volunteers and individuals diagnosed with a psychiatric disorder, comprising both the effects of CBD alone as well as in direct comparison to those induced by ∆9-tetrahydrocannabinol (THC), the main psychoactive component of *Cannabis*.

**Results:** One-ninety four studies were identified, of which 17 met inclusion criteria. All studies investigated the acute effects of CBD on brain function during resting state or in the context of cognitive tasks. In healthy volunteers, acute CBD enhanced fronto-striatal resting state connectivity, both compared to placebo and THC. Furthermore, CBD modulated brain activity and had opposite effects when compared to THC following task-specific patterns during various cognitive paradigms, such as emotional processing (fronto-temporal), verbal memory (fronto-striatal), response inhibition (fronto-limbic-striatal), and auditory/visual processing (temporo-occipital). In individuals at clinical high risk for psychosis and patients with established psychosis, acute CBD showed intermediate brain activity compared to placebo and healthy controls during cognitive task performance. CBD modulated resting limbic activity in subjects with anxiety and metabolite levels in patients with autism spectrum disorders.

**Conclusion:** Neuroimaging studies have shown that acute CBD induces significant alterations in brain activity and connectivity patterns during resting state and performance of cognitive tasks in both healthy volunteers and patients with a psychiatric disorder. This included modulation of functional networks relevant for psychiatric disorders, possibly reflecting CBD’s therapeutic effects. Future studies should consider replication of findings and enlarge the inclusion of psychiatric patients, combining longer-term CBD treatment with neuroimaging assessments.

## Introduction

Recently, there has been a growing interest in cannabidiol (CBD) as a therapeutic substance, due to its putative antipsychotic, anxiolytic and anti-craving effects (Iseger and Bossong, 2015; Rohleder et al., 2016; Batalla et al., 2019). CBD is one of the more than 100 cannabinoids that can be derived from the cannabis plant and is, unlike the main psychoactive compound delta-9-tetrahydrocannabinol (THC), devoid of intoxicating effects (Freeman et al., 2019). Since most conventional treatments in psychiatry, such as antipsychotics and antidepressants, are associated with limited response rates and adverse events that often limit tolerability and adherence (Blessing et al., 2015; Samara et al., 2019), there is an urgent need for developing novel pharmaceutical treatments (Leucht et al., 2013; Blessing et al., 2015; Lally and MacCabe, 2015). In this regard, CBD has been proposed as novel therapeutic compound in several psychiatric disorders, such as psychosis (Iseger and Bossong, 2015; Batalla et al., 2019), anxiety disorders (Blessing et al., 2015), substance use disorders (Chye et al., 2019; Freeman et al., 2020) and autism spectrum disorders (Poleg et al., 2019; Fusar-Poli et al., 2020).

CBD effects are most likely related to the endocannabinoid system (Rohleder et al., 2016), although its precise mechanism of action is not yet fully understood. Animal studies have shown that CBD has no significant affinity with the cannabinoid receptors CB_1_ and CB_2_ (Bisogno et al., 2001; Jones et al., 2010), but may act as an antagonist of both in presence of CB_1_ agonists (Thomas et al., 2007). It has been hypothesized that the antagonistic effects of CBD might be through negative allosteric modulation of the CB_1_ receptor (Laprairie et al., 2015; Rohleder et al., 2016). Other suggested molecular targets include different types of receptors, such as serotonin type 1A (5HT_1A_), peroxisome proliferator-activated receptor gamma (PPARgamma), vanilloid receptor 1 (TRPV1), GPR55, and GPR18 (Pertwee, 2008; Gururajan and Malone, 2016). In addition, CBD has been shown to increase plasma levels of the endogenous cannabinoid anandamide, which was related to its antipsychotic effects (Leweke et al., 2012). Hence, CBD may exert a protective effect on disturbances of the endocannabinoid system, as observed in several psychiatric disorders (Leweke et al., 2007; Morgan et al., 2013; Minichino et al., 2019).

Neuroimaging techniques provide a highly useful insight into the human neural processes involved in the behavioral effects of cannabinoids. An increasing number of neuroimaging studies have been performed to examine the human neural mechanisms underlying the effects of CBD. Although some of these studies have been included in excellent reviews that describe the impact of cannabis on human brain function in a broader context (Martín-Santos et al., 2010; Bhattacharyya et al., 2012a; Batalla et al., 2014; Weinstein et al., 2016), the aim of the current review is to provide a systematic and up-to-date overview of neuroimaging studies that investigated the effects of CBD on human brain function. This includes studies that examined the impact of CBD on brain function of healthy volunteers, comprising both the acute effects of CBD alone as well as in direct comparison to those induced by THC, and studies that investigated the neural substrates of acute CBD effects in patients with a psychiatric disorder.

## Methodology

### Search Strategy

This review was conducted in accordance with the Preferred Reporting Items for Systematic Reviews and Meta-Analyses (PRISMA) statement (Moher et al., 2009). PubMed was searched for neuroimaging studies investigating the impact of CBD on human brain function published until May 2020. See for the exact Pubmed search syntax the Supplementary Methods. References were screened for additional relevant studies.

### Data Inclusion

Titles and abstracts were screened blind for eligibility by two authors (AB and JB). Discrepancies were discussed with a third author (MB). Inclusion criteria were: 1) use of neuroimaging techniques, and 2) administration of CBD to human subjects. Reviews and case reports were excluded.

### Data Extraction

Data extraction included: study information (e.g., title, authors, study design); sample characteristics (mean age, sex, handedness); cannabinoid dose and administration route; time interval between administration and imaging; imaging modality; cognitive task performed during imaging; and degree of sample overlap.

## Results

**FIGURE 1:**
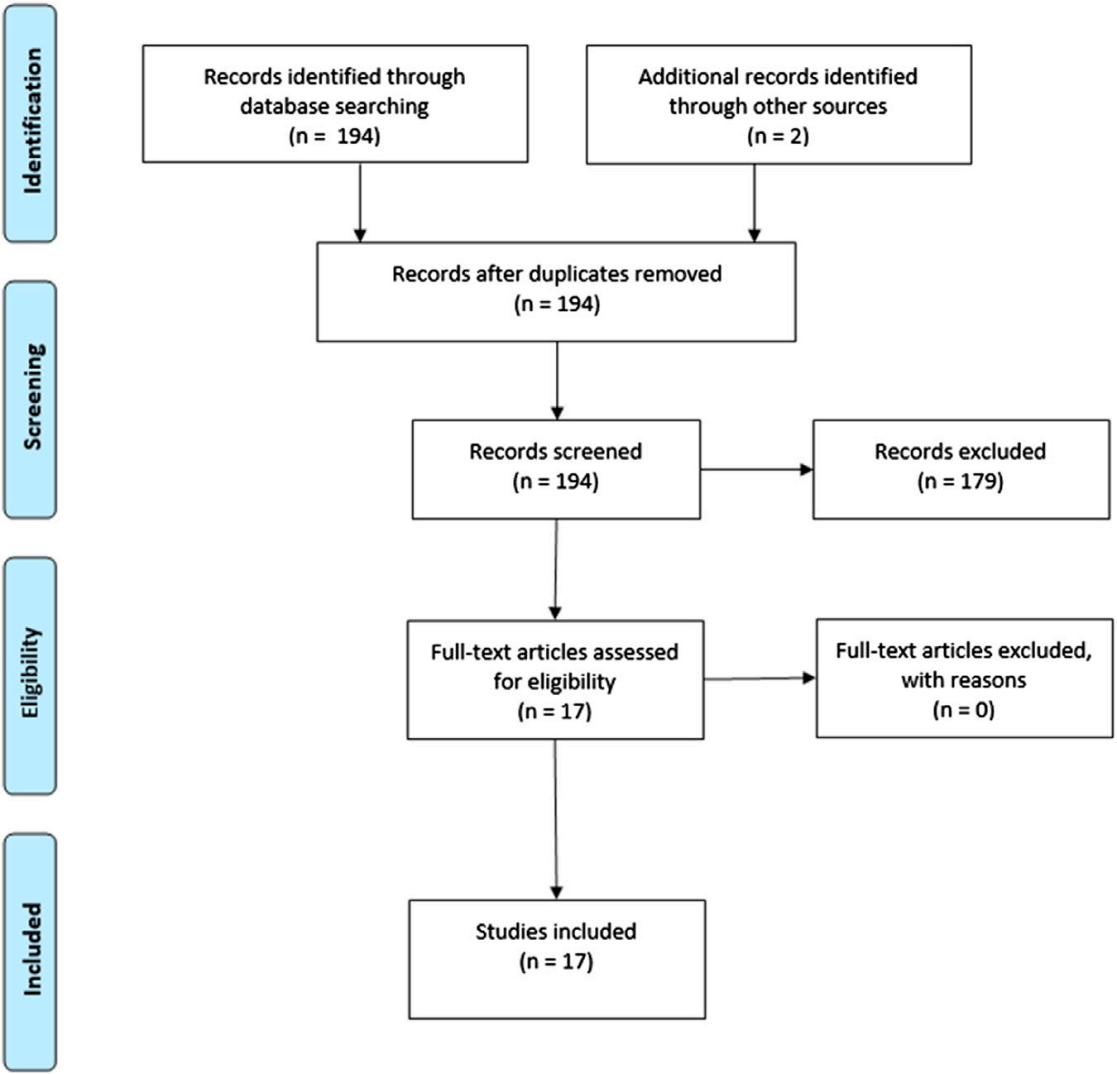
PRISMA flow diagram.

The search strategy yielded 194 studies, of which 15 studies met inclusion criteria. Two studies were found by additional references, resulting in a total of 17 included studies (Figure 1). In total, the current review comprised 115 healthy subjects, 33 individuals at clinical high risk (CHR) for psychosis, 13 patients with a psychotic disorder, 10 patients with anxiety disorder and 17 patients with an autism spectrum disorder.

In healthy subjects, 12 studies reported the acute effects of CBD compared to placebo (Crippa et al., 2004; Borgwardt et al., 2008; Bhattacharyya et al., 2009; Fusar-Poli et al., 2009; Fusar-Poli et al., 2010) or compared to THC (Bhattacharyya et al., 2010; Winton-Brown et al., 2011; Bhattacharyya et al., 2012b; Bhattacharyya et al., 2015; Freeman et al., 2018; Grimm et al., 2018; Wall et al., 2019). In individuals with a psychiatric disorder, five studies assessed the acute effects of CBD compared to placebo (Crippa et al., 2011; Bhattacharyya et al., 2018; Pretzsch et al., 2019; Wilson et al., 2019; O’Neill et al., 2020).

A cluster of eight studies performed different cognitive tasks (i.e., go-no go, verbal learning, emotional processing, visual and auditory processing) using the same sample of healthy subjects (Borgwardt et al., 2008; Bhattacharyya et al., 2009; Fusar-Poli et al., 2009; Bhattacharyya et al., 2010; Fusar-Poli et al., 2010; Winton-Brown et al., 2011; Bhattacharyya et al., 2012b; Bhattacharyya et al., 2015). In addition, the studies of Freeman et al. (2018) and Wall et al. (2019) used an overlapping sample of healthy participants, and those of Bhattacharyya et al. (2018) and Wilson et al. (2019) a similar cohort of CHR individuals. See Tables 1–3 for study characteristics and results of studies included in the current systematic review.

**TABLE 1:** The acute effects of CBD on brain function of healthy volunteers.

First author	Imaging modality	Condition	Image analysis	Study design	HC	M/F	Mean age (SD)	*Cannabis* use	Dose	Route	Imaging findings
Grimm et al. (2018)	3T fMRI	Resting state	Connectivity	DB, PC, Ra, BS	16	NR	NR	NR	600 mg CBD	Oral	↑ Connectivity R putamen with R middle frontal gyrus, BL superior frontal gyrus/paracingulate gyrus, R frontal pole
Bhattacharyya et al. (2015)	1.5T fMRI	Go-no go salience	Connectivity	DB, PC, PR, WS	15	15/0	26.7 (5.7)	<15 times lifetime. Not in the last month	600 mg CBD	Oral	↓ R inferior frontal gyrus with R insula; L anterior lobe of cerebellum; L lingual gyrus; L thalamus; L dorsal striatum with L caudate nucleus body; L inferior frontal gyrus; L dorsal striatum with L anterior cingulate; L medial frontal gyrus; L posterior hippocampus with L parahippocampus; L posterior hippocampus with R parahippocampus; L posterior cingulate; L caudate tail
Bhattacharyya et al. (2012b)	1.5T fMRI	Go-no go salience	Whole brain	DB, PC, PR, WS	15	15/0	26.7 (5.7)	<15 times lifetime. Not in the last month	600 mg CBD	Oral	↓L medial prefrontal cortex
Winton-Brown et al. (2011)	1.5T fMRI	Visual and auditory processing	Whole brain	DB, PC, PR, WS	14	14/0	26.7 (5.7)	<15 times lifetime. Not in the last month	600 mg CBD	Oral	Auditory: BL temporal cortex, BL insula, BL parahippocampal gyri, BL hippocampi; L superior temporal gyrus, L insula, L posterior middle temporal gyrus, L supramarginal gyrus. Visual: R (inferior, middle) occipital lobe, R lingual gyrus, R cerebellum, R cuneus
Fusar-Poli et al. (2010)	1.5T fMRI	Fearful faces	Connectivity	DB, PC, PR, WS	15	15/0	26.7 (5.7)	<15 times lifetime. Not in the last month	600 mg CBD	Oral	Disruption of anterior cingulate cortex–amygdala effective connectivity
Fusar-Poli et al. (2009)	1.5T fMRI	Fearful faces	Whole brain	DB, PC, PR, WS	15	15/0	26.7 (5.7)	<15 times lifetime. Not in the last month	600 mg CBD	Oral	Neutral faces: NS: Intermediate fearful faces: ↓ BL posterior lobe cerebellum. Intensely fearful faces: ↓ L medial temporal region (amygdala and anterior parahippocampal gyrus), anterior and posterior cingulate gyri, R posterior lobe cerebellum
Bhattacharyya et al. (2009)	1.5T fMRI	Verbal learning task	Whole brain	DB, PC, PR, WS	15	15/0	26.7 (5.7)	<15 times lifetime. Not in the last month	600 mg CBD	Oral	NS
Borgwardt et al. (2008)	1.5T fMRI	Go-no go response inhibition	Whole brain	DB, PC, PR, WS	15	15/0	26.7 (5.7)	<15 times lifetime. Not in the last month	600 mg CBD	Oral	↓ L posterior insula, L superior temporal gyrus, L transverse temporal gyrus
Crippa et al. (2004)	99mTc-ECD SPECT rCBF	Resting state	Whole brain	DB, Ra, PC, WS	10	10/0	29.8 (5.1)	<5 times lifetime. Not in the last year	400 mg CBD	Oral	↑ L mediotemporal cortex (parahippocampus, fusiform gyrus). ↓ L. amygdala/hippocampus/hypothalamus, L posterior cingulate cortex

BL, bilaterally; BS, between-subject; CBD, cannabidiol; DB, double-blinded; HC, healthy controls; L, left; M/F, male/female; NB, non-blinded; NC, non controlled; NR, not reported; NS, nonsignificant results; PC, placebo controlled; PR, pseudorandomized; R, right; Ra, randomized; THC, tetrahydrocannabinol; WS, within subject. Grey areas: overlapping samples of subjects.

**TABLE 2:** The acute effects of CBD vs. THC on brain function of healthy volunteers.

First author	Imaging modality	Condition	Image analysis	Study design	HC	M/F	Mean age (SD)	*Cannabis* use	Dose	Route	Imaging findings	Clinical correlations
Wall et al. (2019)	fMRI	Resting state	ROI	DB, PC, R, WS	17	8/9	26,2 (7.1)	<3 times/week and >4 times last year	8 mg THC (Cann-CBD). 8 mg THC +10 mg CBD (Cann + CBD)	Inhalation	Cann + CBD vs. placebo, and Cann-CBD vs. placebo: ↓ mean connectivity in default mode network (defined as positive connectivity with the posterior cingulate cortex). Cann-CBD vs. Cann + CBD: ↓ mean connectivity in salience (defined as positive connectivity with anterior insula)	Cann-CBD: Disruptions in posterior cingulate cortex function in default mode network with subjective feelings of stoned, high, feel drug effect, dry mouth. Frontal pole region in salience network was negatively correlated with feelings of paranoia
Freeman et al. (2018)	fMRI	Auditory processing	ROI	DB, PC, R, WS	16	8/8	26.3 (7.4)	<3 times/week and >4 times last year	8 mg THC (Cann-CBD). 8 mg THC +10 mg CBD (Cann + CBD)	Inhalation	Cann-CBD vs. placebo: ↓ BL auditory cortex, R hippocampus, R ventral striatum, R amygdala. Cann-CBD vs. placebo, and Cann + CBD vs. placebo: ↑ connectivity R ventral striatum with BL auditory cortex (Cann + CBD greater effects)	Cann-CBD and Cann + CBD: ↑ R ventral striatum correlated with pleasure ratings and response to music
Grimm et al. (2018)	3T fMRI	Resting state	ROI	DB, PC, Ra, BS	16	NR	NR	NR	600 mg CBD. 10 mg THC	Oral	CBD > THC: R putamen with frontal pole and paracingulate gyrus	NS
Bhattacharyya et al. (2015)	1.5 T fMRI	Go-no go salience	ROI. Whole brain	DB, PC, PR, WS	15	15/0	26.7 (5.7)	<15 times lifetime. Not in the last month	600 mg CBD. 10 mg THC	Oral	THC > placebo > CBD: R inferior frontal with R parahippocampal gyrus. L posterior hippocampus with L superior-, middle- and inferior frontal gyri, anterior cingulate/medial prefrontal cortex, L precentral gyrus. THC < placebo < CBD: L dorsal striatum with L ventral striatum and with L inferior frontal gyrus. L posterior hippocampus with parahippocampal gyrus	THC: ↓ connectivity between striatum and inferior frontal gyrus with ↑ response latency during standard condition relative to oddball condition
Bhattacharyya et al. (2012b)	1.5 T fMRI	Go-no go salience	Whole brain	DB, PC, PR, WS	15	15/0	26.7 (5.7)	<15 times lifetime. Not in the last month	600 mg CBD: 10 mg THC	Oral	THC > placebo > CBD: R superior, R middle, R inferior and R orbitofrontal gyri. THC < placebo < CBD: L caudate, L putamen, L parahippocampal gyrus, L thalamus, L lingual gyus	THC: ↓ BL caudate head with ↑ severity of psychotic symptoms. ↓ BL caudate head with ↑ response latency to standard stimuli. ↑ R prefrontal cortex with ↑ response latency to standard stimuli
Winton-Brown et al. (2011)	1.5 T fMRI	Visual and auditory processing	Whole brain	DB, PC, PR, WS	14	14/0	26.7 (5.7)	<15 times lifetime. Not in the last month	600 mg CBD. 10 mg THC	Oral	Auditory processing: THC < CBD: R superior and middle temporal gyrus (R side homolog to Wernicke). Visual processing: THC > CBD: L lingual and middle occipital gyriTHC < CBD: BL occipital regions	NS
Bhattacharyya et al. (2010)	1.5 T fMRI	Verbal learning, go-no go, visual and auditory processing, fearful faces	Whole brain	DB, PC, PR, WS	15	15/0	26.7 (5.7)	<15 times lifetime. Not in the last month	600 mg CBD. 10 mg THC	Oral	Verbal learning (recall):CBD > THC: Striatum, anterior cingulate/medial prefrontal, lateral prefrontal. Go-no go: CBD > THC: BL parahippocampus, L insula, caudate. Visual processing: CBD > THC: BL occipital cortex. Auditory processing: CBD > THC: BL lateral temporal cortex: Fearful faces: CBD < THC: L amygdala, fusiform, lingual gyri, lateral prefrontal cortex, cerebellum	THC: ↓ striatum with ↑ severity of psychotic symptoms. ↑ L amygdala with anxiety (STAI). ↑ skin conductance response. CBD: ↓ L amygdala with a trend toward less anxiety (VAMS). ↓ skin conductance response

BL, bilaterally; BS, between-subject; CBD, cannabidiol; DB, double-blinded; HC, healthy controls; L, left; M/F, male/female; NB, non-blinded; NC, non controlled; NR, not reported; NS, nonsignificant results; PC, placebo controlled; PR, pseudorandomized; R, right; Ra, randomized; THC, tetrahydrocannabinol; WS, within subject. Grey areas: overlapping samples of subjects.

**TABLE 3:** The acute effects of CBD on brain function of patients with a psychiatric disorder.

First author	Imaging modality	Condition	Image analysis	Study design	HC	Patients	M/F	Mean age (SD)	*Cannabis* use	Dose	Route	Imaging findings	Clinical correlations
O’neill et al. (2020)	3T fMRI	Verbal learning	ROI	DB, R, PC, WS	19	13 with psychotic disorders, all treated with antipsychotics except for one subject	Pt: 10/5. HC: 11/8	Pt: 27.7 (4.6). HC: 23.9 (4.2)	HC:<10 times lifetime	600 mg CBD	Oral	Encoding: Placebo > control: R inferior frontal gyrus, L inferior and middle frontal gyrus: Placebo < control: L middle frontal gyrus. Placebo > CBD > control: BL inferior frontal gyrus, L middle frontal gyrus. Placebo < CBD < control: L middle frontal gyrus. Recall: Placebo > control: R parahippocampus, R middle and inferior frontal gyri, placebo < control: L parahippocampal gyrus. Placebo > CBD > control: R middle -, R frontal gyrus, R parahippocampal gyrus. Placebo < CBD < control: L parahippocampal gyrus. Connectivity (recall): Placebo > control: Hippocampus with the right caudate head and left caudate body. CBD showed intermediate connectivity relative to placebo and healthy controls between hippocampus and R caudate head, L caudate body, L putamen	↓ activation in inferior frontal gyrus with increase in PANSS score
Wilson et al. ((2019)	3T fMRI	Monetary incentive delay	ROI. Whole brain	DB, PC, R, BS	19	33 CHR, antipsychotic naïve	HC: 11/8. CHR: 17/16	HC: 23.9 (4.2). CHR (CBD) 22.7 (5.1). CHR (placebo) 24.1 (4.5)	HC: NR, CHR (CBD): Current users: 43.8%. CHR (placebo) current users: 41.2%	600 mg CBD	Oral	CHR (placebo) > HC: BL frontal operculae; L insula, parietal operculum; L superior frontal gyri, L inferior frontal gyrus, frontal operculae; L superior temporal gyrus. CHR (placebo) > CHR (CBD) > HC: L insula, parietal operculum; L frontal operculum; L superior frontal gyrus	CHR (placebo): Negative correlation between b-values and mean reaction time difference between salience and neutral conditions. Positive correlation between activation in L insula/parietal opercula and CAARMS positive subscale. HC: Negative correlation b value in L insula/parietal opercula with mean reaction time for salience condition
Pretzsch et al. (2019)	MRS	Resting state	ROI	DB, PC, PR, WS	17	17 with ASD, unmedicated except for two subjects (methylphenidate and sertraline)	34/0	Pt: 31.3 (9.9). HC: 28.5 (6.6)	NR	600 mg CBD	Oral	HC and ASD: ↑ Glx basal ganglia. ↓ Glx dorsomedialprefrontal cortex. HC: ↑ GABA + basal ganglia and dorsomedialprefrontal cortex. ASD: ↓ GABA + basal ganglia and dorsomedialprefrontal cortex
Bhattacharyya et al. (2018)	3T fMRI	Verbal learning	Whole brain	DB, PC, R, BS	19	33 CHR, antipsychotic naïve	HC: 11/8. CHR (CBD): 10/6. CHR (placebo): 7/10	CHR (CBD): 22.4 (5.0). CHR (placebo): 25.4 (5.2). HC: 23.9 (4.1)	CHR: Most more than once a week. HC: <10 times lifetime	600 mg CBD	Oral	Encoding: CHR (placebo) > HC: R middle frontal gyrus, inferior frontal gyrus, insula; L insula/claustrum, inferior frontal gyrus, putamen; R precentral gyrus, postcentral gyrus, inferior parietal lobule; L cerebellum, lingual gyrus. CHR (placebo) < HC: R subcallosal gyrus, caudate head; L anterior cingulate; R caudate tail, posterior cingulate cortex; R precuneus, cuneus. CHR (placebo) > CHR (CBD) > HC: R inferior frontal, middle frontal gyri, insula; L insula, putamen; 3 clusters in precentral gyri; R fusiform gyrus, cerebellum; L cerebellum, fusiform gyrus. CHR (placebo) < CHR (CBD) < HC: L caudate head, putamen, anterior cingulate cortex; R subcallosal gyrus, caudate head; R caudate tail, posterior cingulate cortex; precuneus, R cuneus, fusiform gyrus. Recall: CHR (placebo) > HC: R inferior frontal, middle frontal, precentral gyri, insula; R cuneus, fusiform, lingual gyri, posterior cingulate gyri; L cerebellum, middle occipital, fusiform gyri. CHR (placebo) < HC: Parahippocampal gyrus, midbrain, cerebellum, thalamus; superior temporal, middle temporal gyri; superior transverse temporal gyri; middle frontal gyrus. CHR (placebo) > CHR (CBD) > HC: R inferior frontal gyrus, middle frontal gyrus, insula; R precuneus, cuneus, lingual, middle occipital, fusiform gyri, cerebellum; L cerebellum, fusiform, lingual, inferior occipital gyri. CHR (placebo) < CHR (CBD) < HC: L parahippocampal gyrus, midbrain, cerebellum; L thalamus; L transverse temporal gyrus, superior temporal gyrus; L precentral, cingulate gyri, caudate body	
Crippa et al. (2011)	99mTc-ECD SPECT rCBF	Resting state	Whole brain	DB, PC, R, WS		10 with social anxiety disorder, unmedicated	10/0	Pt: 27.7 (4.6). HC: 23.9 (4.2)	<5 times lifetime. Not in the last year	400 mg CBD	Oral	↓ L parahippocampal gyrus/hippocampus. ↑ R posterior cingulate gyrus	NS

ASD, autism spectrum disorders; BL, bilaterally; BS, between-subject; CBD, cannabidiol; CHR, clinical high risk of psychosis; DB, double-blinded; HC, healthy controls; L, left; M/F, male/female; NB, non-blinded; NC, non controlled; NR, not reported; NS, nonsignificant results; PC, placebo controlled; PR, pseudorandomized; Pt, patients; R, right; Ra, randomized; THC, tetrahydrocannabinol; WS, within subject. Grey areas: overlapping samples of subjects.

### Acute Effects of CBD on Brain Function of Healthy Volunteers

Nine double-blind placebo-controlled studies investigated the acute effects of CBD on brain function of healthy volunteers. One of these studies used Single Photon Emission Computed Tomography (SPECT) measuring regional cerebral blood flow, whereas eight studies applied functional Magnetic Resonance Imaging (fMRI), either at rest or during the performance of a cognitive task (Table 1).

#### Resting State

Two studies investigated the acute effects of CBD during resting state (Crippa et al., 2004; Grimm et al., 2018). Crippa et al. (2004) measured cerebral blood flow using ^99m^Tc-ethyl cysteinate dimer (^99m^Tc-ECD) SPECT imaging in 10 healthy male volunteers using a cross-over design (Crippa et al., 2004). Administration of an oral dose of 400 mg CBD enhanced blood flow compared to placebo in an area consisting of the left parahippocampal and fusiform gyrus. Conversely, CBD attenuated blood flow in the left posterior cingulate cortex and in a cluster comprising the left amygdala, hippocampus, uncus and hypothalamus (Crippa et al., 2004). In another resting state study using fMRI to measure connectivity, 16 healthy male volunteers were given placebo, 10 mg oral THC and 600 mg oral CBD using a cross-over study design (Grimm et al., 2018). The striatum was set as a seed region and a whole brain analysis was performed. CBD increased connectivity between the right putamen and three clusters, situated mainly in the right prefrontal cortex (Grimm et al., 2018).

#### Cognitive Tasks

Seven studies performed different cognitive tasks (i.e., go-no go, verbal learning, emotional processing, visual and auditory processing) using the same sample of 15 healthy volunteers (Borgwardt et al., 2008; Bhattacharyya et al., 2009; Fusar-Poli et al., 2009; Fusar-Poli et al., 2010; Winton-Brown et al., 2011; Bhattacharyya et al., 2012b; Bhattacharyya et al., 2015). The authors explored the acute effects of 600 mg CBD, 10 mg THC and placebo on brain activity measured by fMRI using a double-blind cross-over design. During the sessions, ratings of anxiety (STAI), intoxication (AIS), psychotic symptoms (PANNS) and subjective feelings (VAMS) were obtained. The acute effects of CBD in direct comparison to those induced by THC administration are described in the next section.

A go-no go task was used to investigate brain activity during response inhibition and detection of salient stimuli (Borgwardt et al., 2008; Bhattacharyya et al., 2012b). Under conditions of response inhibition, CBD attenuated brain activity compared to placebo in the left posterior insula, left superior temporal gyrus and left transverse temporal gyrus (Borgwardt et al., 2008). During the presentation of a salient relative to a non-salient stimuli CBD attenuated activity in the left medial prefrontal cortex (Bhattacharyya et al., 2012b). In the same salient relative to non-salient stimuli comparison, Bhattacharyya et al. (2015) conducted connectivity analyses with the inferior frontal gyrus, dorsal striatum and posterior hippocampus set as seed regions. Compared to placebo, a CBD decreased connectivity was found between the following areas: the right inferior frontal gyrus and right insula, left cerebellum, left lingual gyrus and left thalamus; the left dorsal striatum and left anterior cingulate and left medial frontal gyrus; the left posterior hippocampus and right parahippocampus, left posterior cingulate gyrus and caudate tail. Conversely, CBD increased connectivity between the following areas: the left dorsal striatum and left body of the caudate nucleus and left inferior frontal gyrus; left posterior hippocampus and left parahippocampus (Bhattacharyya et al., 2015).

The verbal learning task consisted of an encoding block, where participants had to evaluate whether pairs of words fitted well together, and a recall block, during which participants matched the presented with the previously associated word (Bhattacharyya et al., 2009). CBD modulated activation during encoding conditions in the insula, midtemporal gyrus, lingual gyrus, precuneus and precentral gyrus. During recall, CBD modulated activation in the hippocampus. However, none of these findings were statistically significant (Bhattacharyya et al., 2009).

The emotional processing task consisted of a series of faces, including neutral, intermediate and extremely fearful faces (Fusar-Poli et al., 2009). Relative to placebo, administration of CBD did not alter brain activity during the presentation of neutral faces. During the presentation of intermediately fearful faces, CBD attenuated activity bilaterally in the posterior lobe of the cerebellum. During the processing of intensely fearful faces, CBD attenuated activity in the left medial temporal region (amygdala and anterior parahippocampal gyrus), the anterior and posterior cingulate gyri and the right posterior lobe of the cerebellum (Fusar-Poli et al., 2009). In addition, CBD decreased the number of skin conductance response fluctuations, a physiological measure of emotional response. Moreover, this decrease in skin conductance response covaried with the attenuation of activity in both the left amygdala and the anterior cingulate (Fusar-Poli et al., 2009). Based on these results Fusar-Poli et al. (2010) investigated the connectivity between these two regions in the same sample. Compared to placebo, administration of CBD disrupted connectivity between the left anterior cingulate cortex and the left amygdala while viewing fearful faces (Fusar-Poli et al., 2010).

While listening to neutral words, brain activity was increased during CBD relative to placebo in the bilateral temporal cortex, insula, parahippocampal gyrus and hippocampus (Winton-Brown et al., 2011). Conversely, CBD attenuated activity in the left superior temporal gyrus, insula, posterior middle temporal gyrus and supramarginal gyrus. During visual stimulation, CBD increased activity in the right occipital lobe, lingual gyrus, cerebellum and cuneus (Winton-Brown et al., 2011).

In summary, CBD enhanced fronto-striatal connectivity and decreased limbic activity during resting state, and modulated brain activity showing task-specific patterns during different cognitive paradigms. For example, CBD increased activation relative to placebo in the parahippocampus during auditory processing, and reduced activation in this region during the processing of fearful faces. In addition, CBD decreased connectivity between fronto-limbic regions (i.e., anterior cingulate cortex and amygdala) during the processing of fearful faces and enhanced fronto-limbic-striatal connectivity (i.e., inferior frontal gyrus, dorsal striatum and posterior hippocampus) during salience processing.

### Acute Effects of CBD vs. THC on Brain Function of Healthy Volunteers

Seven fMRI studies investigated the acute effects of CBD in direct comparison to those induced by THC, during resting state or a cognitive task. Some studies analysed regions in the brain where CBD and THC showed opposite activity relative to placebo, whereas others directly compared both substances (Table 2).

#### Resting State

Grimm et al. (2018) conducted a resting state connectivity analysis on 16 healthy volunteers, where the striatum and frontal regions were set as regions of interest. While CBD enhanced frontal-striatal connectivity, THC did not alter this connectivity significantly, possibly due to low THC plasma concentrations during scanning. Direct comparison between the two substances showed that CBD increased connectivity relative to THC between the right putamen and frontal pole and paracingulate gyrus (Grimm et al., 2018).

In a double-blind, pseudo-randomized, within-subject study, Wall et al. (2019) investigated the effects on the resting-state functional connectivity of two strains of inhaled cannabis, containing THC (8 mg) without or with CBD (10 mg), and placebo in 17 occasional cannabis users. Connectivity analyses were performed to investigate the default mode network (defined as positive connectivity with the posterior cingulate cortex), executive control network (defined as negative connectivity with the posterior cingulate cortex) and salience (defined as positive connectivity with the anterior insula). Both strains of cannabis showed a significant reduction in mean connectivity in the default mode network relative to placebo. In the salience network, cannabis containing both THC and CBD caused a significant increase in connectivity compared to cannabis without CBD, but both strains did not differ significantly from placebo. No significant effects were found within the executive control network (Wall et al., 2019). Significant correlations between the subjective measures of feeling the drug’s effect and brain effects were only found after cannabis without CBD was administered. These correlations involved the posterior cingulate cortex region and the frontal pole region (Wall et al., 2019).

#### Cognitive Tasks

Freeman et al. (2018) investigated the acute effects of inhaled cannabis with and without CBD, while they listened to classical music and scrambled sound, using the same sample of occasional cannabis users as described by Wall et al. (2019). Both types of cannabis increased ratings of wanting to listen to music and enhanced sound perception. Inhalation of cannabis without CBD relative to placebo resulted in a dampened response to music bilaterally in the auditory cortex, right hippocampus, right ventral striatum and right amygdala. Cannabis with CBD did not significantly modulate activity relative to placebo or cannabis without CBD. Across all sessions, activation in the right ventral striatum was correlated with pleasure ratings and response to music. Moreover, this region showed an increased functional connectivity with the bilateral auditory cortex during music relative to scrambled sound. Cannabis with CBD had a greater impact on the functional connectivity between these two regions relative to cannabis without CBD (Freeman et al., 2018).

The other series of four studies performed different cognitive tasks (i.e., go-no go, verbal learning, emotional processing, visual and auditory processing) in a double-blind cross-over design of 600 mg CBD, 10 mg THC and placebo, using the same sample of 15 healthy volunteers as described by Borgwardt et al. (2008).

During an emotional processing task, CBD and THC had opposite effects relative to placebo in the left amygdala, fusiform, and lingual gyri, the lateral prefrontal cortex and the cerebellum (Bhattacharyya et al., 2010). The increased activity in the left amygdala following THC administration covaried with the level of anxiety assessed by the STAI, while the attenuated activity after CBD in the amygdala correlated to its anxiolytic effect measured by the VAMS. Opposite effects on skin conductance response fluctuations were also found following the administration of THC compared to CBD (Bhattacharyya et al., 2010).

During the recall phase of a verbal memory task, CBD enhanced and THC reduced brain activity in the striatum (Bhattacharyya et al., 2010). The reduction in the striatum activity after THC administration correlated with the severity of psychotic symptoms. Furthermore, during the recall phase opposite effects were found in a cluster consisting of the anterior cingulate and medial prefrontal cortex and in the lateral prefrontal cortex (Bhattacharyya et al., 2010).

During response inhibition, CBD increased and THC reduced activity in the left insula, left caudate and bilateral parahippocampal gyrus (Bhattacharyya et al., 2010). During a go-no go task, CBD attenuated and THC increased activity in the right superior, middle, inferior and orbifrontal gyri compared to placebo (Bhattacharyya et al., 2012b). Conversely, in left caudate, putamen, parahippocampal gyrus, thalamus and lingual gyrus, activation was attenuated by THC but augmented by CBD (Bhattacharyya et al., 2012b). Bhattacharyya et al. (2015) conducted a connectivity analyses on the same data with the inferior frontal gyrus, dorsal striatum and posterior hippocampus set as seed regions. CBD and THC modulated functional connectivity between these seeds and clusters in the rest of the brain in opposite direction (Bhattacharyya et al., 2015).

During processing of speech, CBD and THC showed opposite effects relative to placebo in the bilateral temporal cortex, whereas opposite effects were found in the bilateral occipital cortex while viewing a visual checkerboard (Bhattacharyya et al., 2010). A direct comparison of CBD and THC effects revealed significantly reduced activity after THC in the right superior and middle temporal gyrus during processing of speech. During visual processing, THC increased activity relative to CBD in the bilateral lingual and middle occipital gyrus, but reduced activity in several other occipital regions. Mixed effects were reported in the cerebellum (Winton-Brown et al., 2011).

In summary, CBD and THC showed dissonant effects during resting state and during several cognitive tasks. During resting state, CBD enhanced connectivity between fronto-striatal regions compared to THC, and cannabis with both THC and CBD increased connectivity within the salience network compared to cannabis without CBD. THC and CBD showed task-specific opposite effects during emotional processing (fronto-temporal), verbal memory (fronto-striatal), response inhibition (fronto-limbic-striatal), and auditory/visual processing (temporo-occipital).

### The Acute Effects of CBD on Brain Function of Patients With a Psychiatric Disorder

Five neuroimaging studies reported the acute effects of CBD on brain function in patients with a psychiatric disorder. Three of these studies used fMRI: two in a similar cohort of individuals at clinical high for psychosis and one in a group of patients with established psychosis. One study used SPECT to investigate cerebral blood flow in patients with social anxiety disorder and one study examined metabolite concentrations using proton Magnetic Resonance Spectroscopy (^1^H-MRS) in patients with autism spectrum disorder (Table 3).

Bhattacharyya et al. (2018) conducted an fMRI double-blind randomized trial on 33 medication-naïve CHR subjects and 19 healthy controls, using the same verbal learning task as described in previous studies (Bhattacharyya et al., 2009; Bhattacharyya et al., 2010). Patients were administered 600 mg CBD or placebo, while healthy controls were not given any drug. During encoding conditions, the group of patients who received placebo (indicative of the at-risk state) showed altered brain activity compared to the healthy control group in clusters involving the frontal gyrus, the insula, claustrum, dorsal striatum, pre- and postcentral gyrus, parietal gyrus, cerebellum, lingual gyrus, subcallosal gyrus, cingulate cortex, precuneus and cuneus. During recall conditions, the group of patients who received placebo showed altered brain activity relative to the healthy control group in clusters comprising the frontal gyrus, insula, cuneus, fusiform, lingual gyrus, posterior cingulate, cerebellum, occipital gyrus, fusiform gyrus, parahippocampal gyrus, midbrain, cerebellum, thalamus and temporal gyrus. A linear comparison across the three groups (patients receiving CBD, patients receiving placebo, and control subjects receiving no drug) revealed several clusters in which CBD showed intermediate activation compared to the placebo and healthy control group. For instance, during encoding, the CBD group showed intermediate activation in clusters encompassing the frontal gyrus, insula, striatum, precentral gyrus, cerebellum, fusiform gyrus, cingulate cortex, subcallosal gyrus and occipital gyrus. During recall, the CBD group showed intermediate activation (relative to the placebo and control group) in clusters comprising the frontal gyrus, insula, striatum, precentral gyrus, cerebellum, fusiform gyrus, cingulate cortex, occipital gyrus, parahippocampal gyrus, midbrain, thalamus and temporal gyrus (Bhattacharyya et al., 2018).

Wilson et al. (2019) conducted a monetary incentive delay task in the same 32 CHR medication-naïve subjects and 19 healthy controls reported by Bhattacharyya et al. (2018). This tasked was used to investigate motivational salience conditions by comparing brain activation during reward and loss relative to neutral anticipation. The group of patients who received placebo showed greater brain activity compared to the healthy control group in clusters encompassing the frontal opercula, insula, parietal operculum, frontal gyri, and temporal gyri. A linear comparison between the three groups revealed intermediate activation in the CBD group (compared to the placebo and control group) in three clusters: the left insula and parietal operculum, left frontal operculum, and left superior frontal gyrus (Wilson et al., 2019).

One fMRI study explored the effects of CBD on patients with established psychosis (O’Neill et al., 2020), where 15 patients on antipsychotic treatment were given 600 mg CBD or placebo in a double-blind, randomized, within-subject design. In this study, 19 healthy participants were scanned but were not given any drugs. During the scanning procedure all participants performed a verbal learning task, the same used in previously described studies (Bhattacharyya et al., 2009; Bhattacharyya et al., 2010; Bhattacharyya et al., 2018). The medial temporal lobe, prefrontal cortex and striatum/*pallidum* were selected as regions of interest and activation patterns as well as a connectivity analysis were performed (O’Neill et al., 2020). Patients after CBD administration showed a trend level towards a greater decrease in median total PANSS score compared to those receiving placebo. Healthy controls scored better on both encoding and recall of the task compared to patients (after CBD or placebo). Patients under placebo showed increased activation compared to controls in the right inferior frontal gyrus and left inferior and middle frontal gyrus during encoding, while having both increasing and attenuating effects in two different clusters in the left middle frontal gyrus. A linear comparison between the three groups showed that patients under CBD treatment had intermediate activation in several clusters located bilaterally in the inferior frontal gyrus, and the left middle frontal gyrus. The two clusters in the right inferior frontal gyrus were similar to clusters found in the placebo vs. control analysis (O’Neill et al., 2020). During recall, patients under placebo showed increased activation relative to healthy controls in the right middle and inferior frontal gyri and right hippocampus, but decreased activation in the left hippocampus. Similar clusters were found in all of these areas such that CBD had intermediate activation relative to the placebo and control group. Patients under placebo condition displayed increased connectivity between the hippocampus and the right caudate head and left caudate body during recall conditions. CBD had intermediate functional connectivity relative to the other two groups in connections between the hippocampus and right caudate head, left caudate body and left putamen (O’Neill et al., 2020).

Crippa et al. (2011) investigated the acute effect of an oral dose of 400 mg CBD in 10 medication-naïve patients with social anxiety disorder, while using _99_mTc-ECD SPECT imaging to measure cerebral blood flow in a within-subject design. Compared to placebo, CBD decreased subjective anxiety and blood flow in a cluster consisting of the left parahippocampal gyrus and hippocampus, but enhanced blood flow in the right posterior cingulate gyrus (Crippa et al., 2011).

Pretsch et al. (2019) investigated the acute effects of 600 mg CBD on 17 patients with autism spectrum disorder and 17 healthy controls. Magnetic resonance spectroscopy was used to measure glutamate and glutamine (Glx) and inhibitory γ-aminobutyric acid and macromolecules (GABA+) levels in two voxels placed in the basal ganglia and dorsomedial prefrontal cortex. Both groups received placebo and CBD. The effect of CBD on Glx levels showed the same pattern in both patients and controls: CBD increased Glx levels relative to baseline in the basal ganglia and decreased Glx levels in the prefrontal cortex. However, the effects of CBD on GABA + levels showed an opposite pattern between groups: GABA + levels in both the basal ganglia and prefrontal cortex increased in the control group after CBD administration but decreased in the patients with autism (Pretzsch et al., 2019).

In summary, acute brain effects after CBD administration were different in patients with a psychiatric disorder compared to healthy controls. In subjects at CHR for psychosis, CBD administration showed intermediate activity in brain areas involved in memory and reward processing compared to placebo and healthy controls. An intermediate activity was also reported in patients with psychosis after CBD administration during a memory task. CBD also modified limbic activity in subjects with social anxiety, and showed similar (glutamate) and opposite (GABA) patterns of metabolite levels in patients with autism compared to healthy controls.

## Discussion

The current review provides a systematic literature overview of studies that investigated the acute effects of CBD on the human brain of healthy volunteers and individuals diagnosed with a psychiatric disorder. Overall, studies in healthy subjects showed that CBD modulated brain activity and had opposite effects when compared to THC in resting state and during several cognitive paradigms (i.e., salience, emotional, memory, response inhibition, auditory/visual processing), following task-specific activation patterns. Acute CBD administration also modulated brain activity in patients with psychiatric disorders by 1) showing intermediate activity compared to placebo and healthy controls in individuals at CHR and with established psychosis, 2) engaging with resting limbic activity in subjects with anxiety disorders, and 3) exhibiting similar (glutamate) and opposite (GABA) metabolite levels in patients with autism compared to healthy controls.

The acute administration of CBD in healthy volunteers modulated networks relevant for psychiatric disorders during resting state and several cognitive tasks, such as fronto-striatal and fronto-limbic circuitry. Fronto-striatal connectivity was enhanced after CBD administration during resting state (Grimm et al., 2018) and activity increased during salience processing (Bhattacharyya et al., 2015). Interestingly, lower functional connectivity in fronto-striatal circuitry has been reported in psychosis, and has been associated with more severe positive symptoms (Fornito et al., 2013). In addition, CBD decreased fronto-limbic activity during resting state (Crippa et al., 2004) and emotional processing (Fusar-Poli et al., 2010). Functional fMRI studies have shown activation of limbic areas in anxiety disorders (e.g., during panic attacks or panic anticipation) (Pfleiderer et al., 2007; Dresler et al., 2013). Based on a mechanistic account of these networks, these findings suggest that CBD might prove useful as treatment by restoring imbalanced networks in these and probably other neurological (Nenert et al., 2020) and psychiatric conditions, such as substance use disorders (Freeman et al., 2020). Regarding the last, converging preclinical and clinical evidence have shown promising effects of CBD on reducing craving, negative affect and motivation for substance use (Chye et al., 2019; Hurd et al., 2019; Freeman et al., 2020; Spanagel, 2020), phenomena associated with fronto-striatal and limbic network disbalances (Koob and Volkow, 2016; Volkow and Boyle, 2018).

Along these lines, CBD also showed opposite effects compared to THC during resting state and several cognitive paradigms in healthy volunteers. It is known that THC has pro-psychotic and anxiogenic properties, particularly evident with high potency cannabis strains (rich in THC) and at high doses (Campeny et al., 2020; Van der Steur et al., 2020). Opposite neurophysiological effects were reported on prefrontal, striatal and limbic areas, which are relevant neural substrates of psychosis and anxiety, and during several cognitive processes, such as salience, verbal memory, response inhibition, emotional processing and auditory/visual processing. Importantly, striatum activity correlated with severity of psychotic symptoms after THC (Bhattacharyya et al., 2010; Bhattacharyya et al., 2012b), and divergent amygdala activity correlated with severity of anxiety after CBD and THC (Bhattacharyya et al., 2010). These opposite brain effects may therefore underlie the neural basis for the antipsychotic and anxiolytic properties of CBD, and suggest that CBD might be able to counterbalance THC induced effects (Colizzi and Bhattacharyya, 2017). However, CBD concentrations needed to offset the effects of THC in healthy individuals are still unclear, as CBD might also have different effects when administered at different doses (Solowij et al., 2019).

Acute CBD administration also affected brain networks of subjects diagnosed with a psychiatric disorder. In individuals at CHR for psychosis, CBD showed intermediate activity compared to patients receiving placebo and healthy subjects in regions involved in reward and salience processing (Bhattacharyya et al., 2018; Wilson et al., 2019). A similar intermediate activity was reported in subjects with established psychosis during a memory task (O’Neill et al., 2020). These findings are consistent with the enhanced activity observed in fronto-striatal regions in healthy subjects after CBD (Bhattacharyya et al., 2015; Grimm et al., 2018). Altogether, these findings suggest that CBD could contribute to normalise disbalanced fronto-striatal activity in patients at CHR or with established psychosis. In addition, Crippa and colleagues showed that CBD reduced cerebral blood flow in (para) limbic areas (i.e., hippocampus, parahippocampal and inferior temporal gyrus) in subjects with social anxiety (Crippa et al., 2011). This is congruent with decreased fronto-limbic activity in healthy individuals reported after CBD (Crippa et al., 2004; Fusar-Poli et al., 2010), and suggest that the anxiolytic effects of CBD may be related to the capacity of this compound to modify brain activity in (para) limbic areas (Crippa et al., 2011). Finally, a spectroscopy study in autism spectrum disorder and healthy controls showed similar glutamate (i.e., increased in basal ganglia, and decreased in prefrontal cortex in both groups) and opposite GABA (i.e., decreased levels in patients and increased in controls in both basal ganglia and prefrontal cortex) effects after CBD administration (Pretzsch et al., 2019). This study adds to preclinical evidence that CBD may modulate the activity of other neurotransmitters, even after a single dose (Crippa et al., 2018). This has implications for the homeostasis of other neurotransmitter systems, such as glutamate, GABA and dopamine. However, the underlying molecular mechanisms explaining the relationship between CBD and other neurotransmitters needs further study.

One of these molecular mechanisms may involve the ability of CBD to directly inhibit the reuptake of anandamide. This endocannabinoid has shown anti-inflammatory activity (Pisanti et al., 2017), and its increase after CBD has been related to antipsychotic effects (Leweke et al., 2012; Rohleder et al., 2016). Because endocannabinoids act as retrograde messengers, it has been hypothesized that increased endocannabinoid concentrations after CBD may attenuate presynaptic release of GABA and glutamate, as well as stabilise dopamine neurotransmission (Gururajan and Malone, 2016). In addition, most of the reported effects after CBD administration occurred in brain areas rich in CB_1_ receptors (Burns et al., 2007). Chronic cannabis use is associated with reductions in endogenous cannabinoids and down-regulation of CB_1_ receptors (Hirvonen et al., 2012; Morgan et al., 2013), while CBD antagonistic effects could be related to modulation of cannabinoid receptors by binding to a distinct allosteric site (Laprairie et al., 2015). Given that CBD may attenuate THC effects, it has also been speculated that CBD may be able to prevent down-regulation of CB_1_ receptors on the long-term, and thus decrease the risk of developing psychosis and/or substance use disorders (Wall et al., 2019). Other possible mechanisms of action of CBD involve its agonist activity towards 5HT_1A_ receptors (Soares and Campos, 2017), partial agonist activity on dopamine D2 receptors (Seeman, 2016), and the activation of vanilloid receptor 1, a non-selective calcium channel, facilitating glutamate pre-synaptic release (Campos et al., 2012).

This review must be read with a series of limitations taken into account. First, included papers often employed different methodologies (e.g., imaging method, route of administration, applied doses), although we used strict inclusion and exclusion criteria for article selection to avoid excessive heterogeneity between studies. For example, whereas most studies administered CBD and THC as individual cannabinoid compounds in separate sessions (Borgwardt et al., 2008; Bhattacharyya et al., 2012b; Grimm et al., 2018), some studies examined the impact of CBD on brain function by comparing effects of cannabis containing THC only to cannabis with both THC and CBD (Freeman et al., 2018; Wall et al., 2019). Regarding differences in applied cognitive paradigms, it is important to note that the described effects on brain activity might depend on the nature of the task used or stimuli presented, as different tasks might provoke distinct brain activity patterns. For instance, whereas memory paradigms may heavily rely on recruitment of temporal and prefrontal areas (Bhattacharyya et al., 2009; Bhattacharyya et al., 2018; O’Neill et al., 2020), emotional and salience processing mainly involve limbic activation (Fusar-Poli et al., 2009; Bhattacharyya et al., 2012b). One final methodological aspect that should be taken into account is that the clinical and brain effects of CBD might be different depending on age and illness progression (Di Marzo et al., 2015; Batalla et al., 2019; Colizzi et al., 2020), or be influenced by the concomitant use of medication (e.g., antipsychotics) or drugs of abuse. However, the within-subject design of the studies where concomitant use of medication or cannabis was allowed probably mitigated these confounding effects (Table 3). Second, most of the included studies used overlapping samples of mainly male healthy subjects or patients with psychiatric disorders to explore the effects of CBD. Although the studies reviewed herein offer a consistent picture indicating that CBD has modulatory effects over neural networks relevant for psychosis, anxiety and addiction, this highlights the need for replication of findings in independent and larger cohorts also including female subjects.

Suggestions can be made for future research on the impact of CBD on brain function. First, because all studies included in the current review examined the acute effects of CBD administration, future research should focus on longer-term CBD treatment of patients with a psychiatric disorder in combination with neuroimaging assessments, in order to elucidate neural substrates underlying the therapeutic effects of CBD. In this respect, two excellent examples of studies nearing completion are 1) 3 weeks CBD treatment of individuals at CHR for psychosis (Institute of Psychiatry, King’s College London), and 2) 4-week add-on CBD treatment of early-onset patients with a psychotic disorder (University Medical Center Utrecht, Netherlands), both in combination with baseline and follow-up functional MRI and ^1^H-MRS techniques. Second, because the clinical response to CBD has been shown to differ between patients (Batalla et al., 2019), future studies could also apply neuroimaging techniques to contribute to identification of those patients that may particularly benefit from CBD treatment.

In conclusion, neuroimaging studies have shown that CBD modulates brain activity and connectivity in neural systems relevant for psychosis and anxiety, possibly reflecting CBD’s therapeutic effects. Future studies should consider replication of findings and enlarge the inclusion of psychiatric patients, combining longer-term CBD treatment with neuroimaging assessments.

## Data Availability Statement

The original contributions presented in the study are included in the article/Supplementary Material, further inquiries can be directed to the corresponding author.

## Author Contributions

AB and JB performed the systematic search and drafted the manuscript, and MB screened potentially eligible publications and critically revised the manuscript for important intellectual content. AB, JB, AP, and MB provided critical revisions for important intellectual content and significantly contributed to the manuscript. All authors have read and agreed to the published version of the manuscript.

## Conflict of Interest

The authors declare that the research was conducted in the absence of any commercial or financial relationships that could be construed as a potential conflict of interest.

## Abbreviations

AIS, intoxication scale; CB_1_, cannabinoid receptor 1; CB_2_, cannabinoid receptor 2; CBD, cannabidiol; CHR, clinical high risk; fMRI, functional magnetic resonance imaging; GABA, γ-aminobutyric acid; GPR18, G protein-coupled receptor 18; GPR55, G protein-coupled receptor 55; Glx, glutamate and glutamine; H-MRS, proton magnetic resonance spectroscopy; 5HT_1A_, serotonin type 1A receptor; PANNS, positive and negative syndrome scale; PPARgamma, peroxisome proliferator-activated receptor gamma; PRISMA, preferred reporting items for systematic reviews and meta-analyses; SPECT, single photon emission computed tomography; STAI, state-trait anxiety inventory; ^99m^Tc-ECD, ^99m^Tc-ethyl cysteinate dimer; THC, tetrahydrocannabinol; TRPV1, transient receptor potential vanilloid 1; VAMS, visual analog mood scales.
