# Supplementary material for: The Impact of Cannabidiol on Human Brain Function: A Systematic Review
Source: Front Pharmacol. 2021 Jan 21;11:618184. doi: 10.3389/fphar.2020.618184 (PMC7858248; doi:10.3389/fphar.2020.618184)
Supplement: Supplementary file 1 [file datasheet1.pdf]

Supplementary Methods

The impact of cannabidiol on human brain function: A systematic review

Albert Batalla, MD, PhD[1]; Julian Bos[1]; Amber Postma[1], MD; Matthijs G. Bossong, PhD[1] *

[1] Department of Psychiatry, UMC Utrecht Brain Center, Utrecht University, Utrecht, the Netherlands

Pubmed search syntax

(((((((((((((("magnetic resonance imaging"[MeSH Terms] OR "neuroimaging"[MeSH Terms]) OR "fMRI"[Title/Abstract]) OR "magnetic resonance"[Title/Abstract]) OR "blood oxygen level"[Title/Abstract]) OR "BOLD"[Title/Abstract]) OR "neuroimaging"[Title/Abstract]) OR "brain imaging"[Title/Abstract]) OR "MRI"[Title/Abstract]) OR "single photon emission tomography"[Title/Abstract]) OR "SPECT"[Title/Abstract]) OR "positron emission tomography"[Title/Abstract]) OR "PET"[Title/Abstract]) OR "MRS"[Title/Abstract]) AND "humans"[MeSH Terms]) AND (("cannabinoids"[MeSH Terms] OR "cannabidiol"[Title/Abstract]) AND "humans"[MeSH Terms])
